# Optoacoustic micro-tomography at 100 volumes per second

**DOI:** 10.1038/s41598-017-06554-9

**Published:** 2017-07-31

**Authors:** X. Luís Deán-Ben, Hernán López-Schier, Daniel Razansky

**Affiliations:** 1 Institute of Biological and Medical Imaging (IBMI), Helmholtz Zentrum München, Neuherberg, Germany; 2 Research Unit Sensory Biology and Organogenesis, Helmholtz Center Munich, Neuherberg, Germany; 3 School of Medicine and School of Bioengineering, Technical University of Munich, München, Germany

## Abstract

Optical microscopy remains a fundamental tool for modern biological discovery owing to its excellent spatial resolution and versatile contrast in visualizing cellular and sub-cellular structures. Yet, the time domain is paramount for the observation of biological dynamics in living systems. Commonly, acquisition of microscopy data involves scanning of a spherically- or cylindrically-focused light beam across the imaged volume, which significantly limits temporal resolution in 3D. Additional complications arise from intense light scattering of biological tissues, further restraining the effective penetration depth and field of view of optical microscopy techniques. To overcome these limitations, we devised a fast optoacoustic micro-tomography (OMT) approach based on simultaneous acquisition of 3D image data with a high-density hemispherical ultrasound array having effective detection bandwidth beyond 25 MHz. We demonstrate fast three-dimensional imaging of freely-swimming zebrafish larvae, achieving 3D imaging speed of 100 volumes per second with isotropic spatial resolution approaching the dimensions of large cells across a field of view exceeding 50mm^3^. As opposed to other microscopy techniques based on optical contrast, OMT resolves optical absorption acoustically using unfocused light excitation. Thus, no penetration barriers are imposed by light scattering in deep tissues, suggesting it as a powerful approach for multi-scale functional and molecular imaging applications.

## Introduction

Microscopy refers to a myriad of methods aimed at resolving structures invisible to the naked eye. The angular resolution of the human eye is estimated to be approximately 1 arcminute, i.e., points separated by less than ~75 μm cannot be distinguished via normal vision at the closest focusing distance (~25 cm)^1^. This resolution barrier can be readily overcome with different microscopic techniques based on electromagnetic radiation^2^, electron beams^3^ or ultrasound^4^. Of particular importance is optical microscopy, which is arguably the most widespread modality in biomedical research owing to its unique ability to non-invasively and dynamically interrogate *in vivo* biology with molecular- and cell-specific contrast. The recently introduced super-resolution techniques have further extended the spatial resolution beyond the optical diffraction limit^5, 6^. Nonetheless, light scattering still imposes hard limits on the penetration depth of optical microscopy into biological tissues^7^.

Optoacoustic (photoacoustic) imaging has emerged as a promising modality capable of high-resolution imaging beyond the penetration limits of optical microscopy^8^. Since optoacoustics can resolve optical absorption contrast acoustically, the spatial resolution typically scales with 1/200 of the imaging depth^9^, enabling microscopic imaging at depths beyond several millimeters. Acoustic-resolution optoacoustic microscopy is usually implemented analogously as acoustic microscopy, i.e., by raster-scanning a spherically-focused ultrasound transducer to form an image. The axial (depth) resolution in this case scales with the acoustic detection bandwidth^10^ and is ultimately limited by acoustic attenuation^11^. On the other hand, the lateral resolution is further conditioned by the numerical aperture of the transducer^12–14^. Numerous implementations have been reported that use point-by-point scanning of focused transducers with detection bandwidths from several MHz up to the GHz range, corresponding to acoustic spatial resolution down to micrometer scales^15, 16^.

The advantages of a raster-scan-based configuration stem from the ease of implementation. Multiple systems for high-resolution mapping of the optical absorption at diffuse light depths have been reported^17–22^. However, techniques employing focused ultrasound detectors are often afflicted by severe out-of-focus^23^ and limited-view^24^ imaging artifacts. The focal zone of the detector also restricts the field-of-view in the depth direction that can efficiently be covered with each scan. Most importantly, implementation of real-time imaging remains challenging with raster scanning approaches, in particular when it comes to three-dimensional (3D) image acquisition. Overall, it has been shown that accurate and fast optoacoustic imaging performance implies simultaneous tomographic acquisition of 3D data using broad angular (tomographic) coverage around the imaged object^25^. In this regard, sampling of data using dense spherical matrix array configurations represents a convenient acquisition geometry, which has demonstrated superb *in vivo* imaging performance in small animals and human studies^26–30^. However, implementation of high-frequency spherical arrays providing detection bandwidths beyond 10 MHz has remained a technological challenge.

Herein, we introduce a novel tomographic design that enables accurate real-time 3D imaging of optical absorption with microscopic resolution, approaching single cell dimensions at depths beyond the limits imposed by light scattering.

## Results

**Figure 1:**
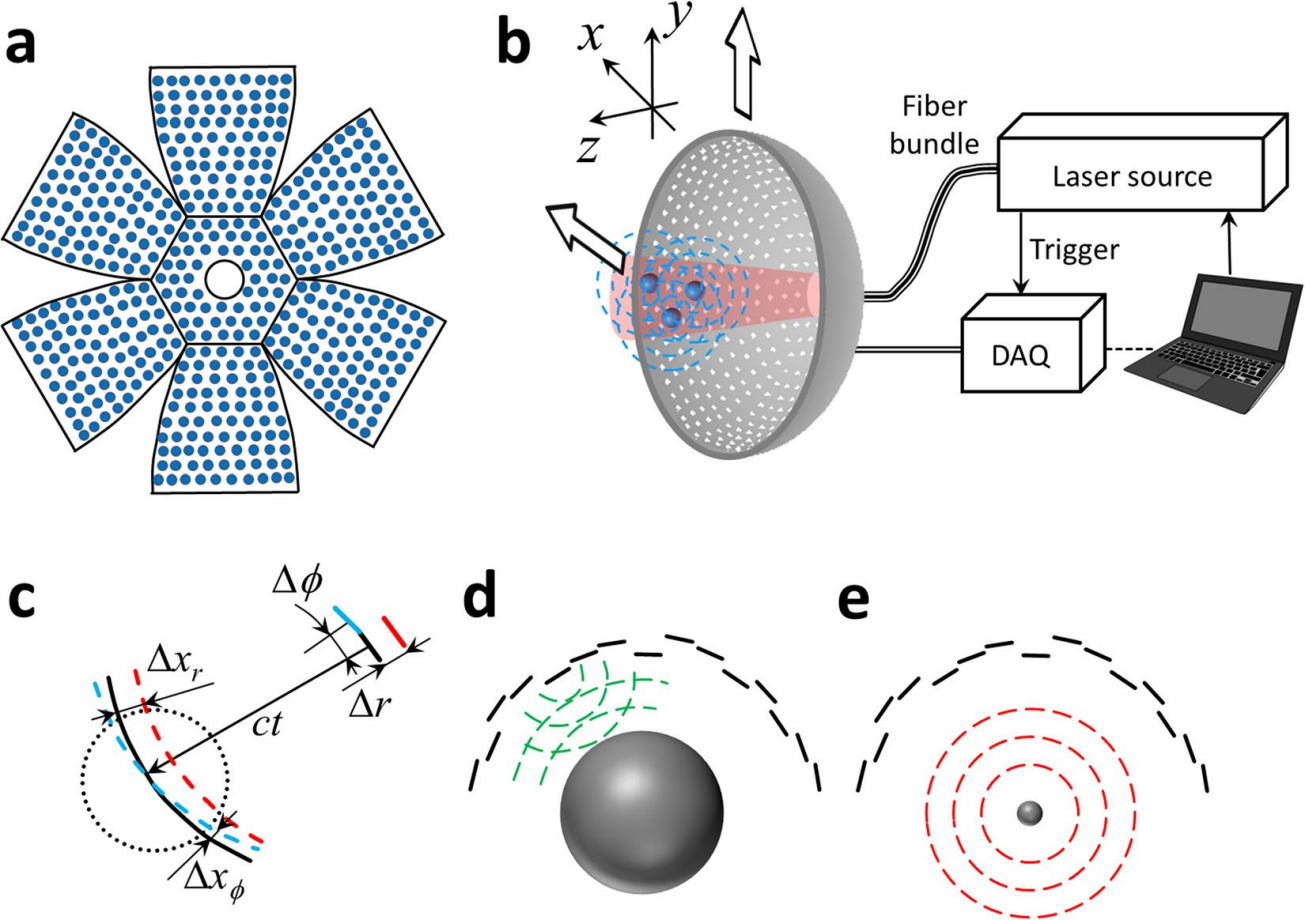
(**a**) Sketch of the high-frequency spherical matrix detection array having an effective detection bandwidth beyond 25 MHz. Distribution of the 510 sensing elements is shown along 7 segments, which are bent to form a hemispherical surface. (**b**) Optoacoustic micro-tomography (OMT) set-up. Optoacoustic responses are excited by illuminating optical absorbers with a short-pulsed laser beam guided through a central cavity of the spherical matrix array. Simultaneous signal detection by all array elements and subsequent tomographic reconstruction render three-dimensional images in real time with isotropic spatial resolution of ~35 µm across an effective field of view >50 mm^3^. The array can be further raster-scanned to render larger image volumes at the expense of temporal imaging resolution. DAQ – Data acquisition system. (**c**) Influence of the radial and lateral errors in the position accuracy of the reconstructed images. (**d**) Calibration of the radial position with pulse-echo time-of-flight measurements using a steel sphere with a radius of 13 mm. (**e**) Time-of-flight calibration using optoacoustic signals generated by a ~20 μm polyethylene microsphere located at the center of the spherical array geometry.

Figure 1 shows the experimental concept of the fast optoacoustic micro-tomography (OMT) based on simultaneous acquisition of 3D image data with a high-density hemispherical ultrasound array having effective detection bandwidth beyond 25 MHz (see online methods for a detailed description). Such implementation is challenged by the need to arrange a large number of high-frequency ultrasound detection elements with sufficient solid angular coverage around the imaged sample, ideally exceeding 180°^25^. Our proposed matrix detection array was designed by arranging 510 piezocomposite elements with 1 mm diameter over 7 planar segments in a “flower” geometry (Fig. 1a). The individual segments were subsequently bent to form a hemispherical surface with 15 mm radius covering an angle of 180° (solid angle 2π). The central segment further has a 3.8 mm diameter aperture for delivery of the excitation light.

**Figure 2:**
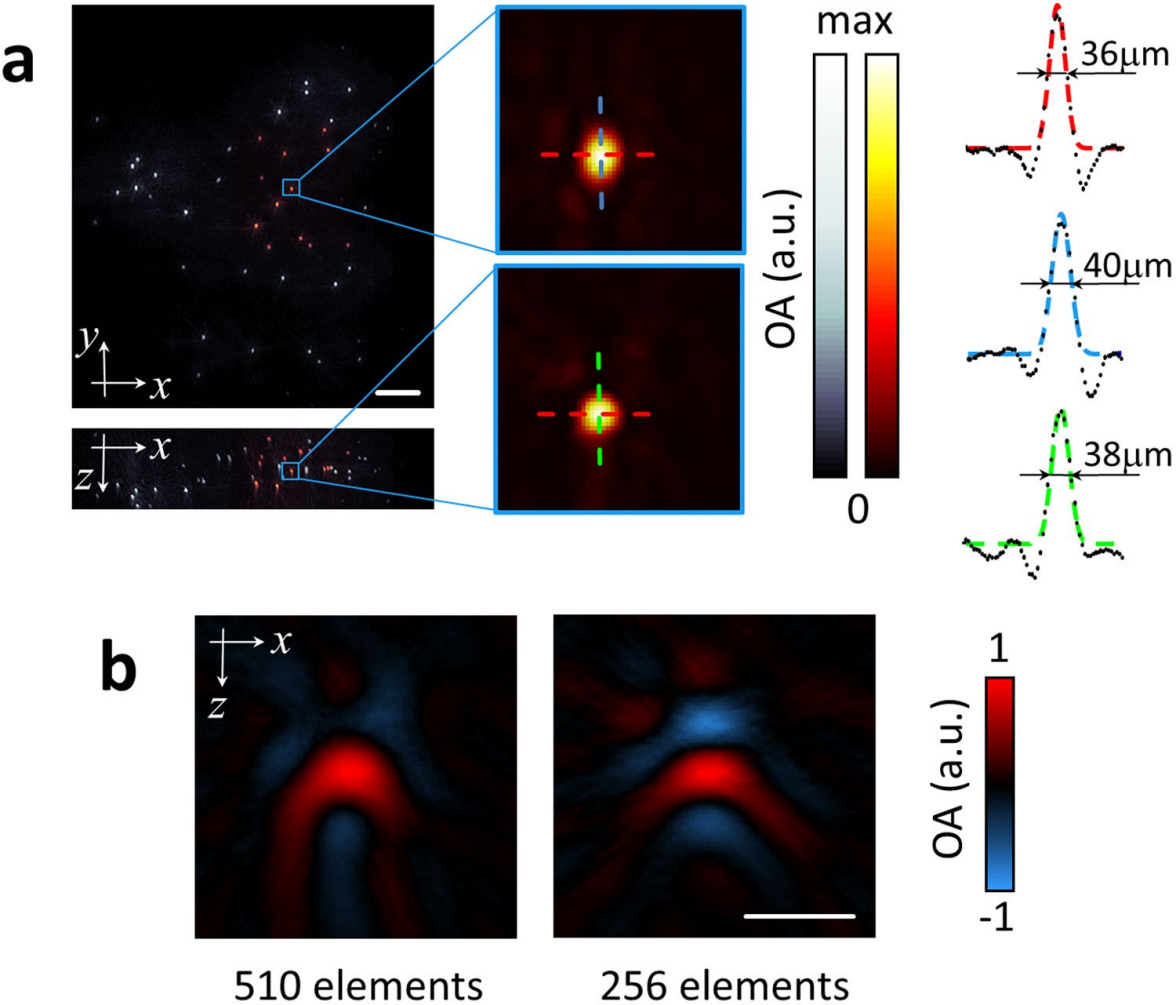
Spatial resolution characterization. (**a**) Maximum intensity projections of the 3D images reconstructed from a single position of the spherical array (‘hot’ colormap) and reconstruction obtained with raster-scanning of the array (‘bone’ colormap). Scalebar – 1 mm. 1D profiles through a single reconstructed sphere (zoom-in is shown in (**a**)) are shown for the x (red), y (blue) and z (green) directions. Colored curves represent Gaussian fits. (**b**) Cross-sections of the reconstructed microsphere located at approximately the center of the transducer array when considering all 510 transducer elements covering 180° angle (left) versus the 256 inner elements covering ~90° angle (right). Scalebar – 100 μm.

Basic resolution characterization was first done with an agar phantom containing ~20 µm diameter absorbing polyethylene microspheres. Figure 2a shows the maximum intensity projections (MIPs) along the z and y directions for the 3D imaging dataset corresponding to a single position of the array (‘hot’ colormap) as well as the entire raster-scanned area (‘bone’ colormap). One may see that the array can effectively render a 3D image from an approximate volume of 50 mm^3^ for each laser pulse. A zoom-in of a microsphere located approximately at the center of the spherical array is also displayed in Fig. 2a. The zoom-in region was reconstructed with a voxel size of 5 × 5 × 5 µm^3^. It is shown that the microsphere was reconstructed with a FWHM <40 µm along all three Cartesian dimensions (one-dimensional (1D) profiles for the x (red), y (blue) and z (green) directions are shown in Fig. 2a). This corresponds to an approximately isotropic resolution of <35 µm estimated as the square difference between the measured FWHM and the actual diameter of the microsphere.

It is also important to highlight that the reconstructed microspheres are not affected by limited-view artefacts due to the broad angular coverage of the array. Limited-view artefacts are often manifested as negative ‘shadows’ in the reconstructed absorbers, which ultimately affect the visibility of elongated structures^24^. As comparison, Fig. 2b shows the lateral cross-section of a reconstructed microsphere in the center of the array when all the 510 detection elements are considered (left, full 180° angular coverage) along with the equivalent image when only the 256 inner elements are taken (right, ~90° angular coverage). As expected, negative image values are much less prominent with the broad 180° angular coverage, although limited detection bandwidth of the transducer elements may further contribute to generation of the negative values in the images.

**Figure 3:**
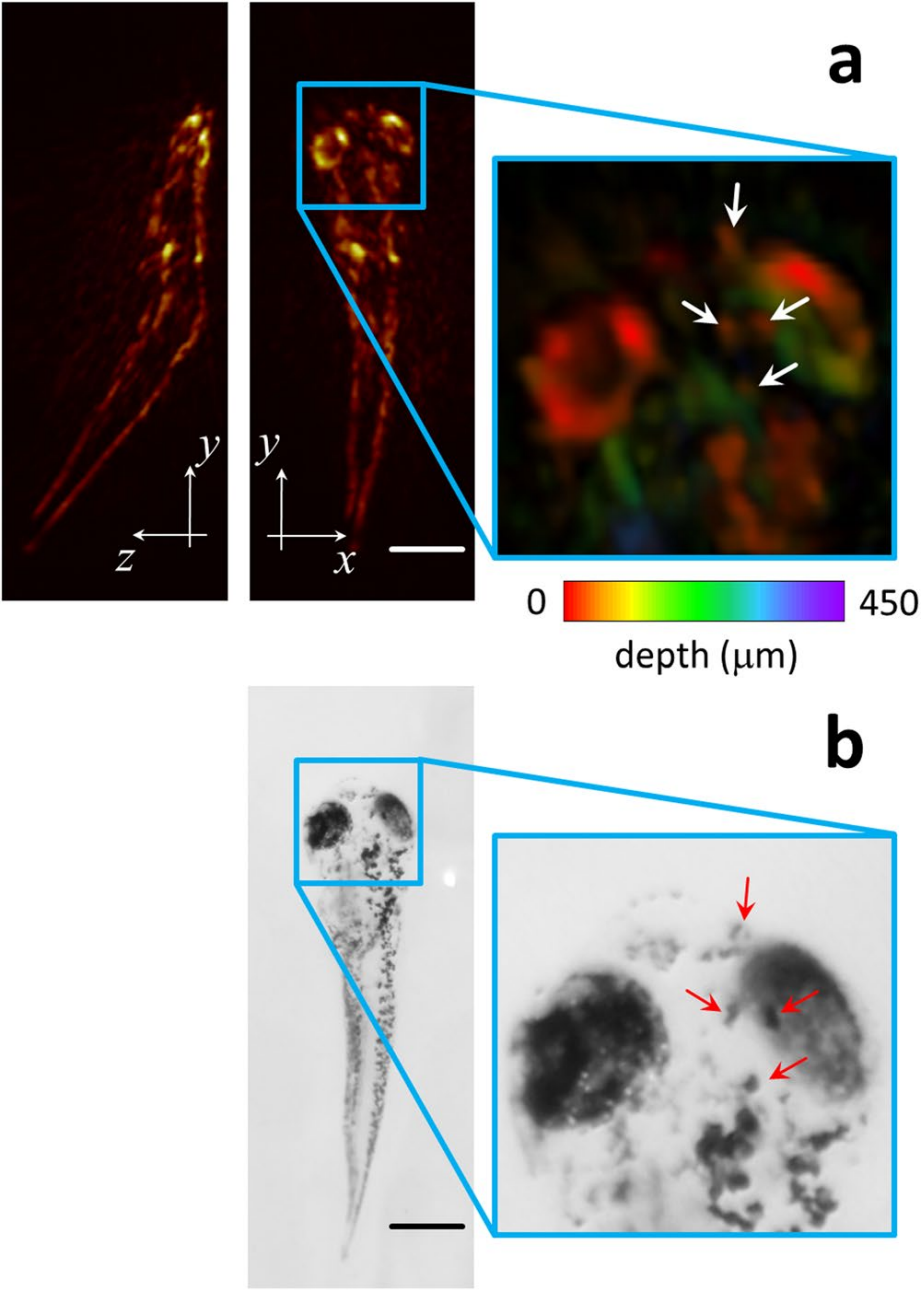
Images of a 6-dpf zebrafish larva *post mortem*. (**a**) Maximum intensity projections of the 3D optoacoustic image (**b**). Corresponding bright field optical microscopy image. Scalebar – 0.5 mm.

A zebrafish larva embedded in an agarose phantom was also imaged *post mortem* in the OMT system. Figure 3 shows the resulting images. Specifically, the top (MIP along the z direction) and lateral (MIP along the x direction) views are displayed in Fig. 3a. Major anatomical features are readily resolvable in the images. The high spatial resolution of the system further enables resolving single cells. Indeed, a closer look at the larva data color-coded for depth reveals individual melanophores containing highly-absorbing eumelanin, whose appearance correlates well with the corresponding bright-field optical microscopy images shown in Fig. 3b.

**Figure 4:**
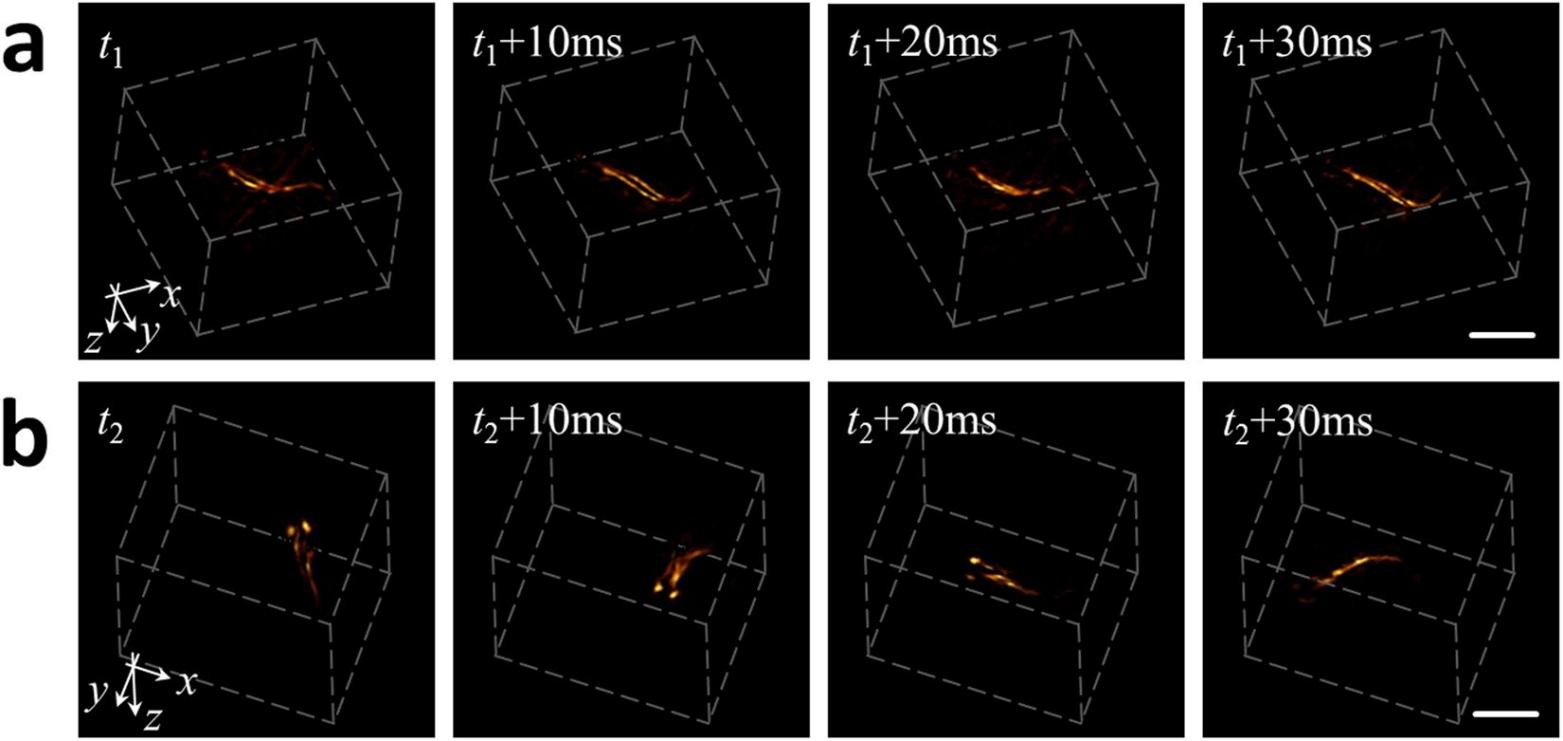
Time-lapse 3D images acquired from a freely swimming zebrafish recorded at a frame rate of 100 volumes per second. The shown sequences correspond to two portions of the movie having smooth (**a**) and abrupt (**b**) movements. Scalebar – 1 mm.

Although zebrafish larvae are optically transparent and thus, in principle, can be efficiently visualized by optical microscopy, OMT has clear advantage in terms of acoustic spatial resolution that is not affected by light scattering in deep tissues as well as the large volumetric field of view and the imaging speed in 3D. This can be better appreciated in Fig. 4, which demonstrates the four-dimensional (4D) visualization capability of a freely-swimming fish. Here, two sequences of four images separated by 10 ms are displayed. In the sequence shown in Fig. 4a the fish swims slowly and its movements can be easily tracked from frame to frame. Note that the system enables constant monitoring of the freely behaving animal in the entire volumetric region of interest determined by the effective field of view of the system. The second sequence (Fig. 4b) shows abrupt movements of the fish with inter-frame motion exceeding 1 mm. Since volumetric tracking at such a high frame rate and field of view is not possible with any existing optical microscopy approach, OMT offers new capacities for imaging biological dynamics with microscopic resolution. The fish motion is best visualized in a movie file available in the on-line version of the journal (Supplementary Video 1).

**Figure 5:**
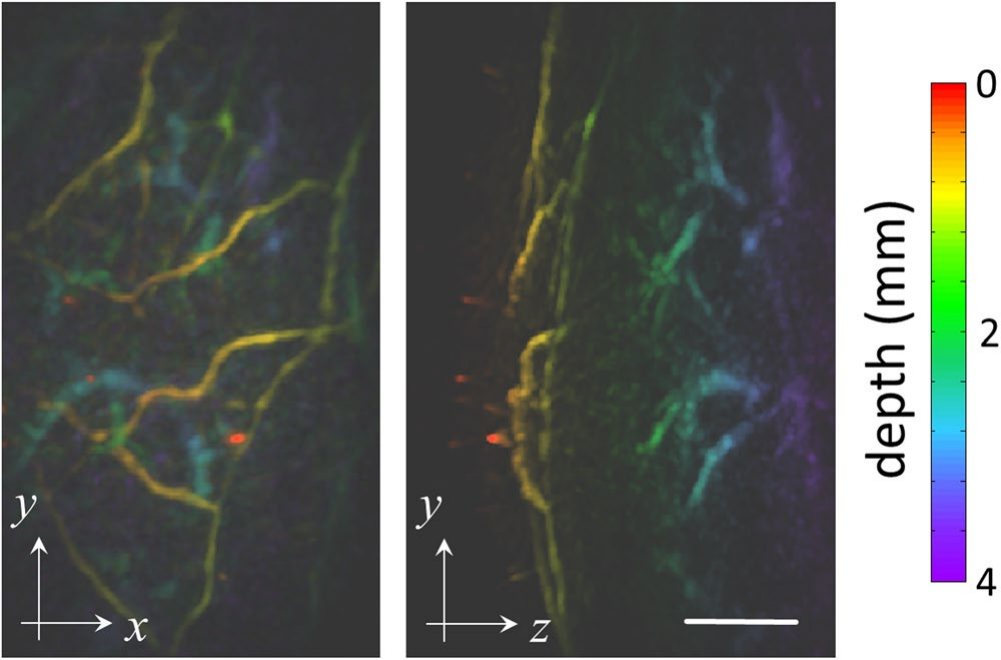
Deep-tissue imaging of mouse microvasculature *in vivo*. Maximum intensity projections of the 3D optoacoustic images are displayed color-coded for depth. Scalebar – 1 mm.

The deep-tissue imaging capabilities of OMT are further illustrated in Fig. 5, where the same hemispherical array was used for rapid 3D scanning of large-scale microvasculature networks in a living athymic nude-Foxn1^nu^ mouse. Specifically, the left lateral side in the abdominal region of the mouse was imaged. Figure 5 displays the MIP images along the *z* and *x* directions respectively. Both images are color-coded for depth. It is shown that vascular structures located at least 4 mm deep into highly absorbing and scattering mammalian tissues can be visualized with the suggested OMT approach. The broad 180° tomographic coverage of the hemispherical array averts image artifacts associated with limited-view acquisition geometries^24^, thus enabling clear visualization of the descending microvasculature propagating at steep angles with respect to the skin surface (Fig. 5, right).

## Discussion and Conclusions

The presented results suggest that the newly developed optoacoustic micro-tomography (OMT) method can acoustically resolve optical absorption contrast with microscopic resolution at a high volumetric frame rate, i.e. in 4D. As opposed to other optical microscopy methods that employ cylindrically or spherically focused light beams, light scattering does not impose penetration barriers for OMT. Indeed, OMT uses unfocused light illumination to image optical absorption contrast with acoustic resolution. This enables the acquisition of 3D tomographic data from the entire imaged volume for each laser pulse, resulting in large fields of view exceeding 50 mm^3^ and volumetric imaging frame rates of 100 Hz, an unprecedented performance among other microscopy methods. OMT may further be extended into imaging spectroscopic dimension by using light at different wavelengths, thus offering five dimensional (5D, real-time multispectral three-dimensional) imaging capabilities^31^.

The presented novel implementation represents an important technological step in terms of the ultrasound sensing performance. The high angular aperture (180°) of the spherical array guarantees broad tomographic coverage, leading to more accurate reconstructions without limited-view artefacts commonly present in systems with lower angular coverage^24, 25^. In addition, previous designs of volumetric optoacoustic tomography have employed spherical array designs at significantly lower frequency range, which resulted in an inferior spatial resolution in the order of 200 µm^32^. These important novel features have enabled here the visualization of microvascular networks, including descending vascular structures, with ~35 μm resolution and penetration depth of 4 mm, thus opening new prospects for imaging of dynamic microcirculation and neovascularization.

Improving the resolution and sensitivity appears to be an important next step for this technology, which would necessitate the development of ultrasonic arrays with larger detection bandwidth. Increasing the temporal resolution can readily be accomplished by employing pulsed lasers with higher repetition frequencies, yet would also imply significantly higher data rates to be matched with faster digital acquisition electronics. On the one hand, increasing the number of ultrasound sensing elements and their detection bandwidth would naturally result in larger fields of view and/or better spatial resolution. However, such developments would again necessitate speeding up data acquisition/processing capacities or otherwise development of sophisticated sparse signal representation and compressed sensing approaches^33–35^. In addition, tomographic reconstruction algorithms must be improved to account for acoustic mismatches and attenuation^11, 36^ as well as for the frequency response of ultrasound transducers^37, 38^.

Indeed, OMT brings the powerful capabilities of 4D and 5D optoacoustics much closer to the single cell dimensions, which may enable a wealth of new applications in imaging of multi-scale biological dynamics. One promising direction is the study of freely behaving animals and organs whose fast motion precludes the application of conventional imaging and microscopy methods lacking fast 3D imaging capabilities in large tissue volumes. Note that the typical fields of view of state-of-the-art intravital microscopy methods are limited to volumes in the 1 mm^3^ range^39, 40^. Another potential biomedical application that may benefit from the newly discovered OMT capabilities is neuroimaging. The recently showcased imaging of genetically encoded calcium indicators (GEGIs) with optoacoustics has set the stage for direct measurements of large-scale neural activity with high spatio-temporal resolution^41^. OMT is ideal for those studies as it can differentiate individual cells with very high temporal resolution, which is sufficient for detecting calcium transients or, potentially, even faster voltage signaling using voltage-sensitive indicators^42, 43^.

In conclusion, the optoacoustic micro-tomography approach introduced in this work is poised to become an important new tool for biomedical research as it brings a new level of spatiotemporal resolution and imaging depth performance not attainable with other optical microscopy modalities.

## Online Methods

### Manufacture of the high-frequency hemi-spherical matrix detection array

The current work aims at devising a new generation of optoacoustic micro-tomography (OMT) system capable of rendering high-resolution (microscopic) 3D reconstructions in real time while also minimizing image artifacts associated with the limited-view tomographic coverage. Such implementation is challenged by the need to arrange a large number of high-frequency ultrasound detection elements with sufficient solid angular coverage around the imaged sample, ideally >180°^25^. In addition, real-time imaging prohibits signal averaging, which imposes demanding requirements on the detection sensitivity of piezoelectric sensing elements (proportional to their size). The proposed matrix detection array was custom-made by Sonaxis SAS (Besancon, France) by arranging 510 piezocomposite elements with 1 mm diameter over 7 planar segments in a “flower” geometry (Fig. 1a). The individual segments were subsequently bent to form a hemispherical surface with 15 mm radius covering an angle of 180° (solid angle 2π). The central segment has a 3.8 mm diameter aperture for light delivery. The electrodes of the piezoelectric elements were sputtered after shaping the segments and the transducer was eventually coated with a thin layer of gold for noise reduction and also to provide a reflecting surface enabling redirecting the scattered light back onto the sample surface.

### Optoacoustic microtomography (OMT) set-up

The entire OMT imaging system is further illustrated in Fig. 1b. Optical excitation is provided with a short-pulsed (<10 ns) optical parametric oscillator (OPO)-based laser (Innolas Laser GmbH, Krailling, Germany) guided via a custom-made 3.8 mm fiber bundle through a central cavity of the transducer array. In this way, an unfocused Gaussian excitation beam with an approximate diameter of 6 mm (at full width at half maximum (FWHM)) is created on the surface of the imaged object placed near the geometrical focus of the spherical array geometry. A high-speed parallel acquisition electronic system that can simultaneously provide time-resolved sampling of all the 510 channels at 125 megasamples per second was further designed (Falkenstein Mikrosysteme GmbH, Taufkirchen, Germany). For each laser pulse, the data from all the channels is transmitted to a personal computer via 1 Gbit/s Ethernet interface for further processing and real-time three dimensional (3D) image reconstruction using a filtered 3D back-projection algorithm^44^. In this way, the volumetric image acquisition is effectively limited to 100 Hz by the pulse repetition rate of the laser. The setup further allows for a raster-scanning of the transducer array in the x-y plane in order to cover larger regions at the expense of temporal resolution (Fig. 1b).

### Calibration of the tomographic detection positions

As the system was designed to provide tomographic reconstructions with spatial resolution in the 30 µm range, location of the individual detection elements in 3D needs to be known precisely. While accurate angular location was ensured by sputtering of the electrodes through a mask to the bent segments, the radial positions of the elements cannot be guaranteed, particularly for an array composed of multiple segments. In fact, any radial inaccuracies in positioning generally have a much larger influence in the achievable resolution than errors in the lateral position of the elements, as depicted in Fig. 1c. For instance, when reconstructing optoacoustic images with the regular back-projection algorithm, a radial uncertainty Δ*r* = 30 µm in the detection location position would readily result in a Δ*x*_*r*_ = 30 µm error in the position of the back-projected arc for a given instant *t* (radius *ct*, being *c* the speed of sound), which corresponds to the error in the position of the reconstructed absorbers and a consequent resolution deterioration. On the other hand, the same position uncertainty in the angular direction (angular uncertanity of Δ*ϕ*.~ 0.1° for the given radius of the spherical array *R = *15 mm) would only result in a maximum error of Δ*x*_*ϕ*_ ~ 5 µm for the effective radius of the imaged field of view of approximately *R*_FV_ ~ 2.5 mm (dotted circle in Fig. 1c).

In order to achieve the best radial position accuracy, calibration was performed with two alternative approaches (Fig. 1d and e). In the first approach, a 13 mm diameter sphere made of steel was positioned at the geometrical center of the array and pulse-echo measurements of the time-of-flight were performed for each element. In the second approach, a ~20 μm diameter polyethylene microsphere (Cospheric BKPMS 20–27 µm) located at the center of the array was illuminated with <10 ns duration laser pulses and the time-of-flight of the optoacoustic responses was measured for each detection element. In both cases, the array was immersed in water, whose temperature was constantly kept at 22 °C during the measurements. The radial position of the elements was then estimated from the time-of-flight measurements by considering the known speed of sound in water for the given temperature^45, 46^.

### Resolution characterization

In order to characterize the resolution and the field of view of the system, a clear agar phantom containing ~20 µm diameter absorbing polyethylene microspheres (Cospheric BKPMS 20–27 µm) was imaged. The wavelength of the laser was set to 720 nm, corresponding to the maximum energy per pulse of 25 mJ within the tunable wavelength range. The microspheres were placed approximately around the center of the spherical array geometry. The array was then raster-scanned along an area of 8 × 8 mm^2^ with a step of 0.5 mm in the x and y directions in order to acquire signals from microspheres distributed across the entire phantom. For each position of the detection array, a 3D image corresponding to a volume of 3 × 3 × 2 mm^3^ (300 × 300 × 200 voxels) was tomographically reconstructed with a graphics processing unit (GPU)-based 3D back-projection algorithm^44, 47^. Prior to reconstruction, the optoacoustic signals were band-pass filtered with cut-off frequencies between 1 and 35 MHz. The resulting images for different positions of the array were superimposed (added up) to render a combined image of a larger region. The field of view was estimated as the volume for which the amplitude of the reconstructed spheres for a given position of the transducer is higher than 50% of the maximum amplitude achieved for a sphere located in the center of the field of view. The field of view estimated in this manner corresponded to an approximately spherical (isotropic) volume of 2 mm radius.

### Biological imaging experiments

The performance of OMT for imaging biological specimens was further tested by 3D recordings of a freely-swimming zebrafish larva and a mouse vasculature *in vivo*. For this, the per-pulse energy density in the imaged region was kept at approximately 20 mJ/cm^2^. All procedures involving animal care and experimentation were conducted in full compliance with the institutional guidelines of the Helmholtz Center Munich and with approval from the Government District of Upper Bavaria.

First, we tested the anatomical imaging capacity of the system. For this, a 6 days-post-fertilization (dpf) wild-type zebrafish larvae was euthanized with 250 mg/l of MS-222 and embedded into a low-melting-point agarose phantom. Since the temporal imaging resolution does not play a crucial role when imaging specimens *post mortem*, the spherical array was raster-scanned along an area of 5 × 2 mm^2^ with a step of 0.5 mm along the x and y directions in order to acquire tomographic data from a larger region. The laser wavelength was set to 720 nm. The acquired signals were also averaged 100 times to improve the signal-to-noise ratio (SNR). Image reconstruction of the scanned area was performed with the same procedure described in section 2D. A three-dimensional median filter with kernel size 3 × 3 × 3 was eventually applied to the reconstructed images.

In the second experiment, two freely-swimming zebrafish larvae were imaged by exploiting the high-frame-rate imaging performance of OMT in 3D. For this, the array was oriented upwards and the entire hemispherical cap detector was filled with agar to guarantee acoustic coupling. The agar block contained a 4 mm diameter cavity filled with water located approximately at the center of the hemispherical array geometry, where the larvae were allowed to swim. The detection array remained stationary during the experiment and the imaging was performed at the highest pulse repetition rate of the laser (100 Hz) without employing signal averaging. The laser wavelength was set to 720 nm. For each laser pulse, a cubic image volume of 64 mm^3^ (180 × 180 × 180 voxels) was reconstructed with the same 3D back-projection algorithm. The optoacoustic signals in this case were band-pass filtering with cut-off frequencies between 1 and 25 MHz for noise reduction. A 3D median filter with kernel size 3 × 3 × 3 was similarly applied to the images.

Finally, a twelve-week-old female athymic nude-Foxn1^nu^ mouse (Harlan Laboratories LTD, Itingen, Switzerland) was imaged to test the deep-tissue imaging capabilities of OMT in mammalian tissues. The mouse was fixed to a holder and immersed in a temperature-controlled water tank, as described elsewhere^48^. The water temperature was maintained at 32 °C during the experiment. The laser wavelength was set to 800 nm. The spherical array was raster-scanned along an area of 5.5 × 3 mm^2^ with a step of 0.5 mm along the x and y directions. The acquired signals were also averaged 100 times to improve the signal-to-noise ratio (SNR). Image reconstruction of the scanned area was performed with the same procedure described in section 2D. In this case, a cubic volume of 96 mm^3^ (160 × 160 × 240 voxels) was reconstructed for each laser pulse. A three-dimensional median filter with kernel size 3 × 3 × 3 was applied to the reconstructed images, which were further normalized with an exponential function to correct for light attenuation^49^.

## Electronic supplementary material

Supplementary Video

Supplementary Information
