# Supplementary material for: Optoacoustic micro-tomography at 100 volumes per second
Source: Sci Rep. 2017 Jul 31;7:6850. doi: 10.1038/s41598-017-06554-9 (PMC5537301; doi:10.1038/s41598-017-06554-9)
Supplement: Supplementary file 2 — Supplementary Information [file 41598_2017_6554_MOESM2_ESM.doc]

A supplementary video file is included with the following article

Title: Optoacoustic micro-tomography at 100 volumes per second

Authors: X. Luís Deán-Ben, Hernán López-Schier and Daniel Razansky

**Supplementary Video 1 Caption.** Image sequence of a freely-swimming 6 days-post-fertilization zebrafish acquired at 100 frames per second for 720 nm illumination wavelength. The movie is displayed at 25 frames per second, i.e., slowed down by a factor of 4 with respect to the real fish motion.
